# Antenatal corticosteroids for management of preterm birth: a multi-country analysis of health system bottlenecks and potential solutions

**DOI:** 10.1186/1471-2393-15-S2-S3

**Published:** 2015-09-11

**Authors:** Grace Liu, Joel Segrè, A Metin Gülmezoglu, Matthews Mathai, Jeffrey M Smith, Jorge Hermida, Aline Simen-Kapeu, Pierre Barker, Mercy Jere, Edward Moses, Sarah G Moxon, Kim E Dickson, Joy E Lawn, Fernando Althabe, Working Group for the UN Commission of Life Saving Commodities Antenatal Corticosteroids

**Affiliations:** 1 Antenatal Corticosteroids Working Group of the UN Commodities Commission, Cambridge, MA, USA; 2 Antenatal Corticosteroids Working Group of the UN Commodities Commission, Oakland, CA, USA; 3 UNDP/UNFPA/UNICEF/WHO/World Bank Special Programme of Research, Development and Research Training in Human Reproduction (HRP), Department of Reproductive Health and Research, World Health Organization, 20 Avenue Appia, 1211 Geneva 27, Switzerland; 4 Department of Maternal, Newborn, Child & Adolescent Health, World Health Organization, 20 Avenue Appia, 1211 Geneva 27, Switzerland; 5 Jhpiego, 1615 Thames St., Baltimore, MD, 21231, USA; 6 University Research Co., LLC, 7200 Wisconsin Avenue, Suite 600, Bethesda, MD 20814, USA; 7 Health Section, Programme Division, UNICEF Headquarters, 3 United Nations Plaza, New York, NY 10017, USA; 8 Institute for Healthcare Improvement, 20 University Road, Cambridge, MA 02138, USA; 9 Gillings School of Global Public Health, University of North Carolina at Chapel Hill, 135 Dauer Drive, Chapel Hill, NC 27599, USA; 10 MaiKhanda Trust, House number 14/56 Off Presidential Drive - Area 14, Private Bag B437, 265 Lilongwe, Malawi; 11 Maternal, Adolescent, Reproductive and Child Health (MARCH) Centre, London School of Hygiene and Tropical Medicine, London, WC1E 7HT, UK; 12 Saving Newborn Lives, Save the Children, 2000 L Street NW, Suite 500, Washington, DC 20036, USA; 13 Department of Infectious Disease Epidemiology, London School of Hygiene and Tropical Medicine, London, WC1E 7HT, UK; 14 Institute for Clinical Effectiveness and Health Policy (IECS), Dr. Emilio Ravignani 2024, Buenos Aires, C1414CPV, Argentina

**Keywords:** Preterm, maternal, newborn, neonatal, antenatal corticosteroids, dexamethasone, gestational age, scale-up, health systems

## Abstract

**Background:**

Preterm birth complications are the leading cause of deaths for children under five years. Antenatal corticosteroids (ACS) are effective at reducing mortality and serious morbidity amongst infants born at <34 weeks gestation. WHO guidelines strongly recommend use of ACS for women at risk of imminent preterm birth where gestational age, imminent preterm birth, and risk of maternal infection can be assessed, and appropriate maternal/newborn care provided. However, coverage remains low in high-burden countries for reasons not previously systematically investigated.

**Methods:**

The bottleneck analysis tool was applied in 12 countries in Africa and Asia as part of the *Every Newborn *Action Plan process. Country workshops involved technical experts to complete the survey tool, which is designed to synthesise and grade health system "bottlenecks", factors that hinder the scale up, of maternal-newborn intervention packages. We used quantitative and qualitative methods to analyse the bottleneck data, combined with literature review, to present priority bottlenecks and actions relevant to different health system building blocks for ACS.

**Results:**

Eleven out of twelve countries provided data in response to the ACS questionnaire. Health system building blocks most frequently reported as having significant or very major bottlenecks were health information systems (11 countries), essential medical products and technologies (9 out of 11 countries) and health service delivery (9 out of 11 countries). Bottlenecks included absence of coverage data, poor gestational age metrics, lack of national essential medicines listing, discrepancies between prescribing authority and provider cadres managing care, delays due to referral, and lack of supervision, mentoring and quality improvement systems.

**Conclusions:**

Analysis centred on health system building blocks in which 9 or more countries (>75%) reported very major or significant bottlenecks. Health information systems should include improved gestational age assessment and track ACS coverage, use and outcomes. Better health service delivery requires clarified policy assigning roles by level of care and cadre of provider, dependent on capability to assess gestational age and risk of preterm birth, and the implementation of guidelines with adequate supervision, mentoring and quality improvement systems, including audit and feedback. National essential medicines lists should include dexamethasone for antenatal use, and dexamethasone should be integrated into supply logistics.

## Background

Each year, an estimated 15 million infants are born premature, representing over one in ten live births [1]. Complications from prematurity are the leading global cause of deaths in children under five [2], with over one million annual deaths [3], particularly from pulmonary immaturity and resultant respiratory distress syndrome (RDS) [4,5]. A preventive approach, first demonstrated to be effective in 1972, is the administration of antenatal corticosteroids (ACS) to mothers at risk of imminent preterm birth in order to stimulate fetal lung maturation [6]. Subsequent trials have established the safety and efficacy of ACS for women at risk of imminent preterm birth, with a meta-analysis of 21 studies finding a roughly one-third reduction in risk of neonatal death [4]. A subgroup analysis of the four trials conducted in middle-income countries (MICs) found a further reduction of around 50% in mortality, suggesting possible greater benefit in lower-resource settings [7].

**Figure 1:**
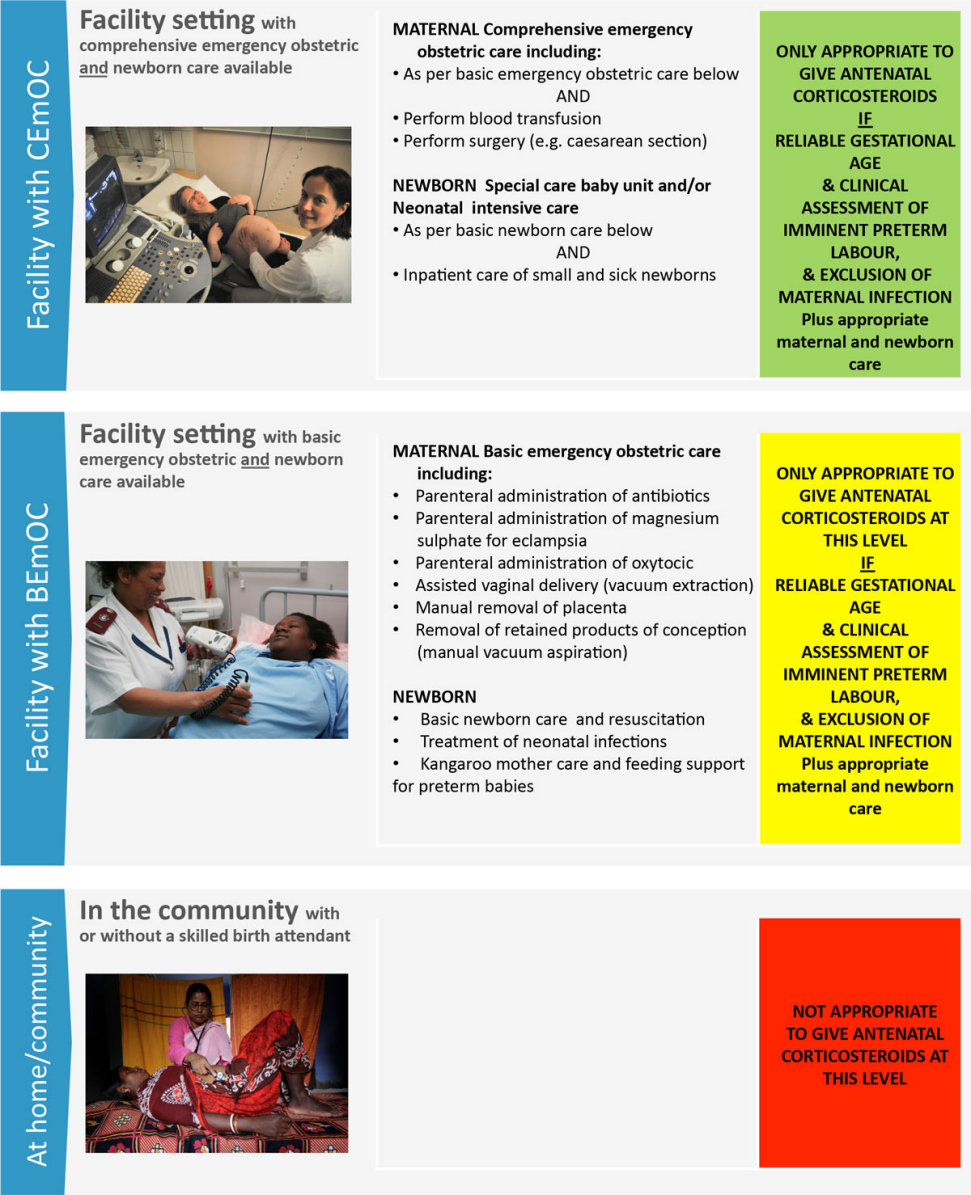
**Levels of care for the safe administration of antenatal corticosteroids for the management of preterm labour**. Facility with comprehensive emergency obstetric and newborn care image source: Mai Simonsen/Save the Children. Facility with basic emergency obstetric and newborn care image source: Chris Taylor/Save the Children. In the community image source: Parth Sanyal/Save the Children

Reducing the burden of preterm birth requires effective maternal care including comprehensive obstetric care (with caesarean section, if needed [8]), and specific care for the preterm newborn [9-11]. ACS administration is a critical component of this care. A recent large, six-country study (Antenatal Corticosteroids Trial, or ACT) extending ACS to community and primary care settings with lower level workers, found adverse outcomes in neonatal mortality, stillbirth and maternal infection, underlining the importance of trained health professionals able to accurately assess gestational age and provide ACS in hospital settings with adequate supportive care [12]. Currently, organisations including the new WHO guidelines on interventions to improve preterm birth outcomes [13] recommend a single course of ACS (dexamethasone or betamethasone, 24 mg administered by intramuscular injection in divided doses) to mothers less than 34 completed weeks of gestation, with risk of imminent preterm birth (anticipated within the subsequent 7 days) [15-20]. Safe and effective use of ACS depends on the accuracy of gestational age assessment, correct diagnosis of imminent preterm birth, and adequate maternal and newborn care (Figure 1). ACS is contraindicated for women with chorioamnionitis.

There has been rapid adoption of ACS use in high-income countries (HICs) since the mid-1990s [20], with coverage rates of over 90%. Yet coverage appears to remain low in low- and middle-income countries (LMICs), where 99% of all neonatal deaths occur [21]. Available estimates suggest that coverage varies greatly in LMIC facilities but is generally low even at the highest levels of care (Table 1). Increased safe and effective use of ACS has the potential to save an estimated 214,300 newborns each year with use for those births already occurring in hospitals with the appropriate package of linked care [22].

**Table 1:** Existing estimates of antenatal corticosteroid coverage using World Bank income groupings.

*Country*	*Coverage estimate (%)*	*Indicator*	*Year*
**42 countries with 90% of child deaths**	5	**All preterm births**	2000 [38]

	***In facility***		

**WHO Multi-country Survey** 7 low income countries 19 middle income countries	27 58	Eligible women 26-34 weeks at facilities with >1,000 deliveries per year and capacity for caesarean section	2014 [39]
**75 Countdown countries** 34 low income countries + 40 middle income countries	41	Preterm births in secondary and tertiary facilities	2014 [40]
**Cameroon (middle income country)**	10	Facility-based RH providers using ACS	2005 [41]
**Brazil (middle income country)**	4	Eligible women 28-33^+6 ^weeks in public maternal hospitals in Rio de Janeiro	1999 [42]
**Ecuador (middle income country)**	35	Eligible women 24-34^+6 ^weeks in influential reference hospitals in capital cities	2010 [43]
**El Salvador (middle income country)**	55		
**Uruguay (high income country)**	71		
**Indonesia (middle income country)**	8	Eligible women <34 weeks in tertiary and district hospitals	2008 [44]
**Malaysia (middle income country)**	28		
**Philippines (middle income country)**	7		
**Thailand (middle income country)**	74		

ACS is only one part of obstetric and preterm birth management [8] and should be part of initiatives to increase institutional birth rates and improve antenatal and intrapartum care coverage, quality and equity. Effectiveness and safety also critically require adequate neonatal care including thermal care, breastfeeding, resuscitation [11], kangaroo mother care [10], and availability of inpatient care of small and sick newborns [9].

Low coverage of ACS has been variously attributed to lack of guidelines, prescribing authority, provider awareness or skills, drug availability, and patient access to appropriate facilities [23]. However, the health system barriers to increased uptake of ACS have not previously been systematically examined.

This paper presents the results of a systematic multi-country analysis of barriers to uptake of ACS for preterm birth. In this analysis, we aim to identify priority health system building blocks facing common and critical bottlenecks to scaling up this life-saving intervention. We additionally discuss policy and programmatic implications and recommend priority actions drawn from the survey responses, existing evidence, and programme experience.

Objectives of this paper are as follows:

1. Use a 12-country analysis to explore health system bottlenecks affecting the scale-up of ACS.

2. Present the solutions to overcome the most significant bottlenecks including insights from respondent countries, literature review and programme experience.

3. Discuss policy and programmatic implications and propose priority actions.

## Methods

This study used quantitative and qualitative research methods to collect information, assess health system bottlenecks and identify solutions to scale-up of maternal and newborn care interventions in 12 countries: Afghanistan, Cameroon, Democratic Republic of Congo (DRC), Kenya, Malawi, Nigeria, Uganda, Bangladesh, India, Nepal, Pakistan and Vietnam. The methodology has been discussed in detail previously and in paper 1 of this supplement [24,25].

### Data collection

The maternal-newborn bottleneck analysis tool was developed as part of the *Every Newborn *Action Plan (ENAP) process to assist countries in identifying context-specific bottlenecks to the scale-up and provision of maternal and newborn health interventions across seven health system building blocks [25]. The tool was utilised during a series of national consultations supported by the global *Every Newborn *Steering Group between July 1^st ^and December 31^st ^2013 (see Additional file 1). The workshops for each country included participants from national ministries of health, United Nations (UN) agencies, the private sector, non-governmental organisations, professional bodies, academia, bilateral agencies, and other stakeholders. For each workshop, a facilitator, orientated on the tool, coordinated the process and facilitated groups to reach consensus on the specific bottlenecks for each health system building block. This paper, third in the series, is focused on antenatal corticosteroids for the management of preterm birth.

### Data analysis methods

We graded bottlenecks for each health system building block using one of the following options: not a bottleneck (=1), minor bottleneck (=2), significant bottleneck (=3), or **very **major bottleneck (=4). We first present the grading in heat maps according to the very major or significant health system bottlenecks as reported by all 12 countries, then by mortality contexts (neonatal mortality rate (NMR) <30 or NMR ≥30 deaths per 1000 live births) and by region (countries in Africa and countries in Asia). We developed a second heat map showing the specific grading of bottlenecks for each health system building block by individual country. Survey responses were analysed for common specific bottlenecks, defined to be those reported by at least 3 countries. Where the same specific bottleneck was reported under more than one building block, it was categorised in accordance with the relevant survey question, or under the building block where most countries reported the specific bottleneck.

Finally, we categorised context-specific solutions for scaling up under the subcategories of corresponding bottlenecks within each health system building block.

## Results

Eleven out of twelve country teams submitted their responses to the ACS questionnaire. Cameroon, Democratic Republic of the Congo (DRC), Kenya, Malawi, Nigeria, Uganda, Bangladesh, Nepal, and Vietnam returned national-level responses. Pakistan provided subnational data from all provinces, Gilgit-Baltistan, and Azad Jammu and Kashmir, excluding two tribal territories. Sindh province, Pakistan, did not provide a grade for community ownership and partnership. India returned data from three states: Andhra Pradesh, Odisha, and Rajasthan. Rajasthan state completed the questionnaire and listed specific bottlenecks, but did not provide ratings for any building block. India responded to the earlier version of the questionnaire with fewer questions. Afghanistan, the twelfth country, returned national-level survey data without responses to the ACS portion and therefore is not included in our analysis.

**Figure 2:**
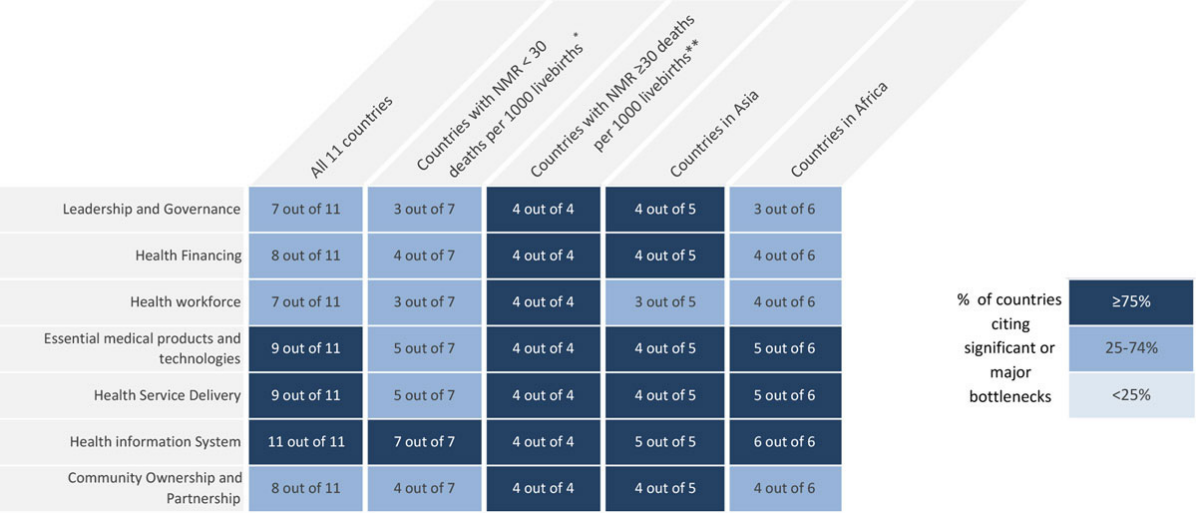
**Very major or significant health system bottlenecks for antenatal corticosteroids for management of preterm birth**. NMR: Neonatal Mortality Rate. *Cameroon, Kenya, Malawi, Uganda, Bangladesh, Nepal, Vietnam. **Democratic Republic of Congo, Nigeria, Afghanistan, India, Pakistan. See additional file 2 for more details.

An overview of grading for the health system building blocks, for all countries and by mortality setting and geography, is shown in Figure 2. More building blocks were graded as having significant or very major bottlenecks in countries with higher NMR; all four countries with NMR ≥30 (DRC, Nigeria, India, and Pakistan) reported very major or significant bottlenecks across all seven health system building blocks. Health system building blocks were graded poorly by a comparable proportion of countries in Africa and Asia, except for leadership and governance, more commonly reported as a very major or significant bottleneck by African respondents.

**Figure 3:**
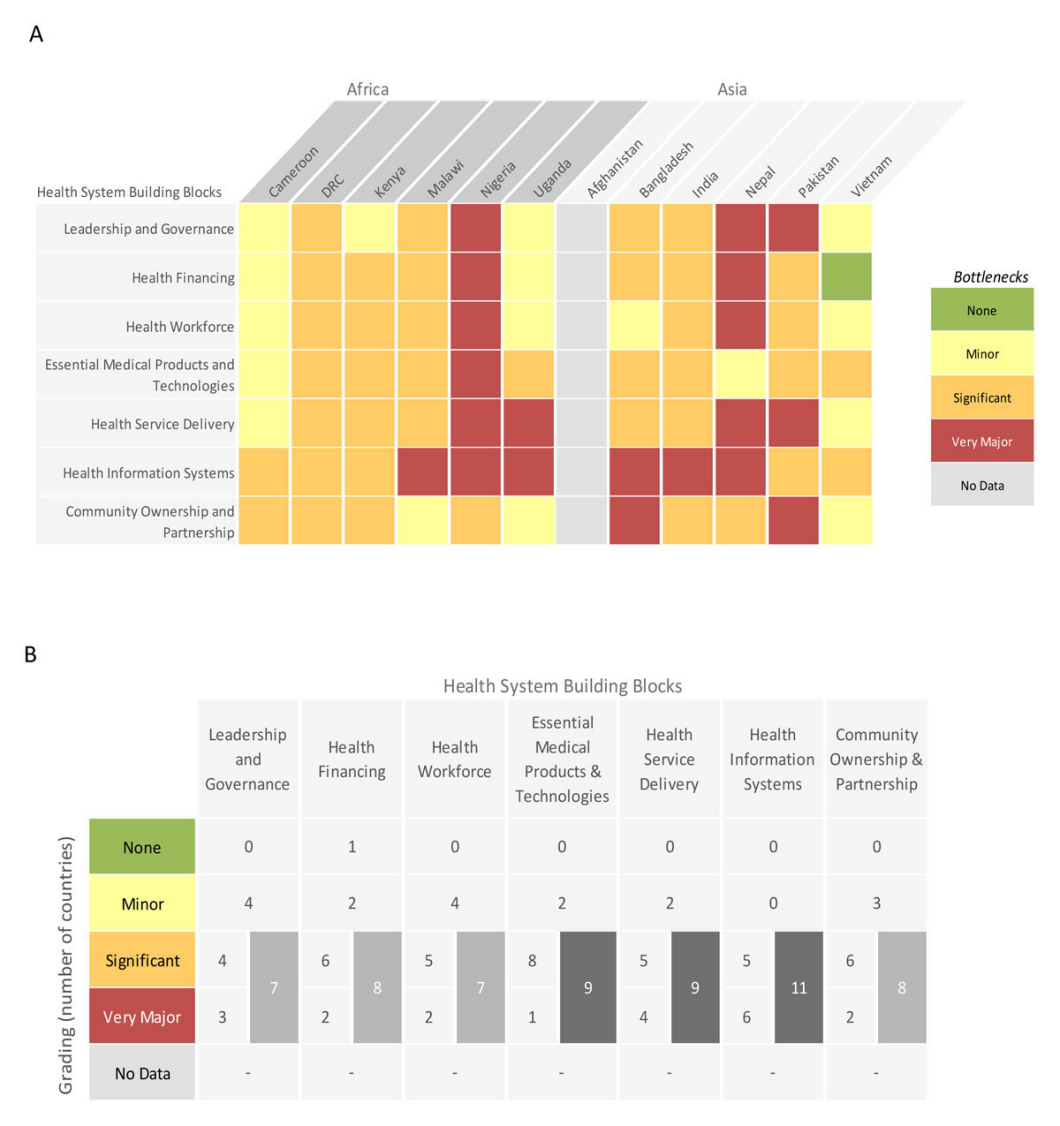
**Individual country grading of health system bottlenecks for antenatal corticosteroids for the management of preterm birth**. Part A: Heat map showing individual country grading of health system bottlenecks for antenatal corticosteroids for the management of preterm birth. Part B: Table showing total number of countries grading significant or major for calculating priority building blocks. DRC: Democratic Republic of the Congo. See Additional file 2 for more details.

Building block grading for each respondent country is summarised in Figure 3. Very major or significant bottlenecks were reported by at least 75% of country teams (at least 9 of 11) in three building blocks: health information systems (all countries), essential medical products and technologies (9 of 11 countries) and health service delivery (9 of 11 countries).

Table 2 summarises priority actions proposed by country teams to address specific bottlenecks to ACS scale-up. These solutions are grouped by health system building block. Common bottlenecks (reported by at least 3 countries) to scale-up of ACS are summarised in additional file 2 (table S1), along with underlying causes where reported.

**Table 2:** Country-recommended priority actions for addressing common bottlenecks to scaling up antenatal corticosteroids.

Health system building block	Sub-category	Priority actions
Leadership and governance	*Policy and guidelines*	• Develop, update and disseminate the national policy on prevention and management of preterm labour; this should include policy on ACS use • Advocate for newborn health to be made as a priority • Develop national clinical protocols and guidelines on management of preterm labour

Health financing	*Funding/budget allocation*	• Increase funding / budget allocation for newborn care • Advocate and lobby for more funding from partners in newborn health
	*Cost of antenatal corticosteroids*	• Assess financial implications and develop financial policy for supplies and services to deliver this intervention to beneficiaries • Include antenatal corticosteroids as part of the free MNCH policy • Scale up obstetric kits by including relevant newborn drugs such as antenatal corticosteroids

Health workforce	*Policy restriction for prescription and administration*	• Authorise all skilled births attendants to prescribe and administer ACS
	*Job descriptions/job aids*	• Develop and disseminate job aids
	*Shortage of qualified staff*	• Recruit and train competent health providers • Develop an electronic database to track training activities and identify needs • Ensure staffing norms are in line with WHO recommendations
	*Competency and training*	• Strengthen competency based pre-service and in-service training, and on job training to capture use of ACS by health providers for fetal lung maturation • Include ACS training in skilled birth attendant evaluation

Health service delivery	*Service availability*	• Develop and disseminate national guidelines in health facilities • Establish follow-up visits to monitor availability and use of ACS in all health facilities
	*Quality of care*	• Establish a supportive supervision and mentoring mechanism with a reward system; • Regular integrated monitoring visits to ensure compliance to protocols; • Integrate ACS use in clinical audits and reviews;
	*Referral systems*	• Involve all stakeholders to improve infrastructure for timely referral (road network) • Build district-level capacity to monitor appropriate use of ambulances and ensure adequate maintenance

Essential medical products and technologies	*Procurement policy*	• Include ACS in national essential medicines list with appropriate indication (fetal lung maturation)
	*Drug availability*	• Develop and disseminate policy in health facilities to enhance procurement
	*Logistics management information systems (LMIS)*	• Estimate needs based on number/estimate of preterm birth load at health facilities • Build staff capacity for logistics management

Health information system	*Data collection and reporting*	• Define indicator(s) for tracking ACS use and incorporate into national system • Strengthen monitoring mechanisms such as regular monitoring and evaluation visits, documentation through use of program data. • Conduct regular review meetings on data management at all levels

Community ownership and participation	*Knowledge / awareness*	• Conduct integrated community maternal and newborn education and campaigns in local languages • Improve community awareness on newborn health by adding newborn information to maternal awareness documents, campaigns, media, etc.
	*Community participation / engagement*	• Strengthen community leaders and male involvement through innovative approaches; • Strengthen functioning of existing community units with nationwide community awareness initiatives on newborn health • Health facilities to come up with innovative ways of involving men e.g. set aside time for couples during antenatal visits and time for adolescents
	*Demand for preterm birth care*	• Scale up tribal empowerment project to address socio-cultural barriers to newborn care • Utilise community radio and mobile applications

### Leadership and governance bottlenecks and solutions

Leadership and governance was graded as a very major or significant bottleneck by 7 of 11 country teams (Figure 2), representing a larger proportion of Asian countries (4 of 5 countries in Asia, compared to 3 of 6 countries in Africa). The most commonly cited specific bottleneck was inadequate guidelines on ACS use, cited by 9 of 11 country teams. Of these, 5 country teams (4 in Asia) reported no clear guidelines on ACS for management of preterm birth, and 4 country teams (all in Africa) reported available guidelines, which were either outdated or not disseminated.

Proposed solutions included development (or update) and dissemination of national guidelines and protocols on prevention and management of preterm labour, including ACS. Updated WHO guidelines will be useful to inform national guidelines.

### Health financing bottlenecks and solutions

Health financing was graded as a very major or significant bottleneck by 8 of 11 country teams. The most commonly cited specific bottleneck was insufficient funding, reported by 9 country teams, with 8 indicating a lack of priority or policy as the underlying cause.

Proposed solutions included increased advocacy and leadership, particularly with funding and budget allocation, for preterm birth prevention, management and care including ACS.

### Health workforce bottlenecks and solutions

Health workforce was graded as a very major or significant bottleneck by 7 of 11 country teams. All country teams questioned reported a shortage of health workers especially in higher cadres, inadequate training, and inadequate supervision or mentoring. India did not provide an answer to the question on health worker shortages.

Proposed solutions included integration of ACS into pre-service and in-service training, development and dissemination of job aids, and consideration of expanded prescription authority for more cadres of health workers.

### Essential medical products and technologies bottlenecks and solutions

Essential medical products and technologies was graded as a very major or significant bottleneck by 9 of 11 country teams. All 11 country teams cited as a specific bottleneck the lack of inclusion of ACS on the national essential medicines list (NEML) for fetal lung maturation, though all included dexamethasone for other indications. Of 8 country teams reporting inadequate procurement or distribution of ACS, 6 cited the lack of policy or NEML listing for fetal lung (or other lack of alignment between supply chain policy and ACS recommendations) as a direct cause of lack of integration into supply chain system. Additionally, no respondent country included ACS in quantification and forecasting, and none reported having an indicator for ACS drug availability.

Proposed solutions included listing of ACS drugs on NEMLs for a fetal lung maturation indication, development and dissemination of guidelines, and software to support needs-based forecasting and procurement.

### Health service delivery bottlenecks and solutions

Health service delivery was graded as a very major or significant bottleneck by 9 of 11 country teams. All country teams reported having inadequate or non-existent quality monitoring and improvement systems, and 9 country teams noted a lack of alignment between ACS prescribing authority and health worker cadres providing care for women at risk of preterm birth. Additionally, 7 countries cited delays due to referrals as a specific bottleneck. Table S3 and S4 of the Additional file 2 summarises levels of care where ACS is allowed and health worker cadres permitted to prescribe and/or administer ACS, as reported by country teams.

Proposed solutions included development and dissemination of guidelines across facilities, creation of supervision and mentoring systems, integration into clinical audits and reviews, and strengthened referral systems.

### Health information system bottlenecks and solutions

Health information system was identified as the most problematic building block, with all 11 country teams reporting very major or significant bottlenecks. Responses to health information systems questions revealed an almost complete absence of defined and standardised indicators for ACS use, with the exception of Nepal and one province in Pakistan, where country teams reported that ACS use was tracked on partographs. No countries reported having an indicator for ACS drug availability. Moreover, no countries included ACS use in clinical audits or perinatal reviews.

Proposed solutions included creation of indicator(s) for ACS use and integration of ACS data into record-keeping systems and regular reviews.

### Community ownership and partnership bottlenecks and solutions

Community engagement was graded as a very major or significant bottleneck by 8 of 11 country teams. The most commonly cited specific bottlenecks were lack of awareness and initiatives targeted at raising awareness of preterm birth risks and management (9 countries), and the absence of an accessible facility or transportation to reach a facility where ACS could be administered (9 countries).

Proposed solutions included leveraging existing community outreach infrastructure, such as community groups, opinion leaders, and media.

## Discussion

This systematic analysis of ACS use in 11 high-burden countries, which together account for over half of maternal and newborn deaths, has identified commonly experienced health system bottlenecks to scaling up ACS for management of preterm birth. A comprehensive approach to preterm birth should include strategies for prevention as well as management; however, the menu for high-impact, evidence-based preterm birth prevention is currently limited [26]. In high-income settings, reductions in the burden of preterm birth can largely be traced to improved care of preterm infants, which has yet to achieve major traction or wide-scale use even for simpler care such as feeding support, kangaroo mother care and infection prevention and treatment [27]. There is also considerable scope for impact through management of preterm birth, including ACS which is highly effective when provided by adequately trained health professionals in hospital settings where adequate maternal and neonatal follow-up and support are possible.

The important principle of "do no harm" and potential risks of ACS have been highlighted recently through the ACT trial. The benefits of ACS are dependent on high coverage in preterm babies <34 weeks; benefit is not expected or is marginal after 34 weeks, and risks increase close to term. Targeting based on gestational age and more accurate diagnosis of imminent preterm birth are therefore key aspects of effective, safe use as underscored in WHO guidelines. In addition, ACS alone cannot be a magic bullet; the preterm infant still requires a minimum of supportive care including warmth and feeding support, and if <32 weeks gestation is more likely to require oxygen therapy and respiratory support. Approximately half of the births in the ACT trial occurred at home or low-level primary care facilities [12].

Despite variation across the 11 countries that responded to the survey, the greatest barriers were consistent, highlighting three priority health system building blocks--health information systems, health service delivery and essential medical products and technologies--with the most critical common bottlenecks. Drawing from country programme experience, and evidence from literature (Table 2), this paper outlines potential solutions for programme managers and policymakers facing similar barriers.

### Health information systems priority actions

Data on ACS coverage, use, and outcomes including safety were absent in nearly all countries. To enable continuous and quantitative assessment of ACS programmes, appropriate indicators need to be defined, and these data integrated into existing health information systems in order to allow tracking and comparison at facility, district, and national levels. ACS coverage data are one of the priority indicators identified in the Every Newborn Action Plan [28]. Coverage data are lacking and should also aim to assess false positives (women treated whose babies are born after 34 completed weeks).

**Figure 4:**
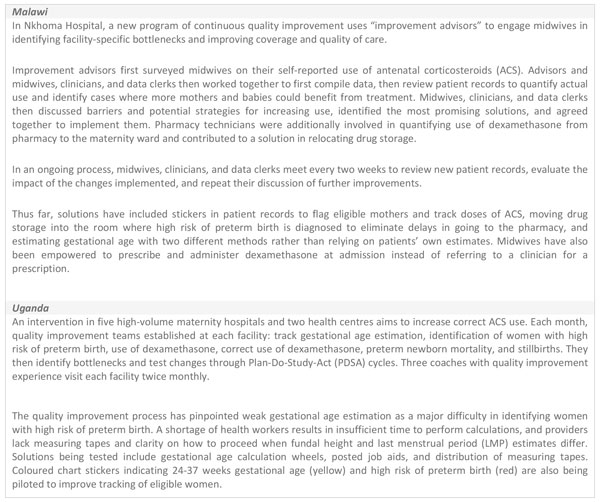
**The use of continuous quality improvement for antenatal corticosteroid use in Malawi and Uganda**. ACS: antenatal corticosteroids. PDSA: Plan-Do-Study-Act

Use of data for quality improvement would be aided if ACS were systematically included in safe childbirth checklists, partographs, clinical audits, and perinatal death reviews [29]. Data are also needed on process such as logistics and stock out. Two case studies of in-country implementation programmes provide examples of data collection and recordkeeping systems for ACS use, and how audit and feedback mechanisms using these records can be harnessed to improve quality of care for the management of preterm birth (Figure 4).

### Health service delivery priority actions

Estimates suggest ACS coverage in LMICs is generally low even in tertiary facilities (Table 1), consistent with the poor grading of health service delivery among respondent country teams. All 11 countries in this analysis are among the 75 Countdown to 2015 countries with an estimate of 41% (weighted average) coverage of preterm births in secondary and tertiary facilities. Eight of 11 countries were also included in the WHO Multicountry Survey estimate of coverage in high-volume facilities; these were DRC (16%), Kenya (32%), Nigeria (30%), Uganda (27%), India (69%), Nepal (20%), Pakistan (63%), and Vietnam (52%). These estimates are likely to be higher than the national average as the survey methodology sampled only large facilities from the capital city and two randomly selected provinces. National population coverage with ACS is likely to be much lower than facility coverage estimates, as a large proportion of births take place outside of high-volume facilities or outside any facility. In the 11 countries, institutional delivery rates (in any facility) ranged from 33% in Bangladesh to 92% in Vietnam, according to data compiled by UNICEF in 2015 [30].

Available evidence supports focusing scale-up efforts in facilities with the capacity for gestational age assessment and diagnosis of women at risk of imminent preterm birth, as well as ongoing support for preterm infants and well-functioning maternity wards [4]. The recent study (ACT) which extended ACS to include non-hospital facilities in six countries (including Kenya, India, and Pakistan) found increased perinatal mortality as well as increased rates of presumed maternal infection [12]. The specific cause(s) of newborn mortality were not ascertainable, but service delivery challenges included limited ability to estimate gestational age and accurately diagnose high risk of preterm birth. As a consequence, ACS was under prescribed for preterm and early preterm babies likely to benefit from treatment and overprescribed to babies born at or near term, when ACS may have increased risks.

With clear evidence for use in hospitals in HICs with adequate neonatal support and reason for caution at lower-level facilities in LIC settings, programmatic scale-up is best focused on higher-level and high-volume facilities, where resources can also be used to produce significant impact cost-effectively and also improve targeting and safety tracking. Increased population coverage will then depend on increased and timely identification and referral of at-risk women, both in lower-level facilities and in homes. Systemic improvements are needed to encourage institutional deliveries with skilled birth attendants and to strengthen referral systems, while guidelines and training at lower levels of care will be critical to building capacity for timely identification. Expanded care such as a pre-referral dose may also improve care by allowing a longer time for ACS to take effect. However, such steps must be considered cautiously by each country based on the capacity of lower-level facilities to provide adequate care to ensure safety.

#### Human resource and skills for service delivery

Within hospitals, coverage is often limited by inadequate numbers of physicians or other providers present and also adequately trained to assess gestational age, diagnose high risk of preterm birth, and authorised to prescribe and administer ACS. The majority of countries reported both a shortage of health workers at higher cadres and a mismatch between provider cadres allowed to prescribe ACS and those cadres likely to be caring for women at risk of preterm birth (Table S2, additional file 2).

Expanded prescribing authority for midwives providing care to pregnant women could greatly increase the capacity of hospitals to manage preterm birth with ACS. However, any change in policy must be considered based on the capability to correctly diagnose conditions, which lead to preterm birth and provide adequate supportive care to both mother and baby. Currently WHO only recommends administration of ACS by doctors and advanced level associate clinicians and recommends against use by nurses and auxiliary nurses. Prescription and administration of ACS by non-advanced associate clinicians has not been evaluated by the WHO due to lack of rigorous evaluation of this question [31]. The WHO recommends cautious consideration of ACS administration by midwives and auxiliary nurse-midwives (ANMs) in LMICs with shortages of physicians-those settings described by respondent countries. Consideration of expanded prescribing authority should be made in the context of rigorous research [31]. In the absence of prescribing authority, health workers providing care to mothers still have an important role in identifying potential risk of preterm birth and ensuring rapid and safe referral.

All countries reported a lack of training in assessing gestational age, recognition of high risk of preterm birth and in management of preterm birth using ACS as well as inadequate supervision and mentoring systems. In-service training and increased support, both for cadres authorised to prescribe and administer ACS and for cadres involved in identification of risk and referral, are also critical to any scale-up effort.

#### Guidelines implementation and quality improvement

For any policy to reach every mother and every newborn, clear guidelines and adequate training must reach all relevant cadres of health worker and levels of care. As indicated by the country teams' responses to leadership and governance questions, it is critical both to develop clear guidelines and to ensure active dissemination. An active model of dissemination should ideally integrate systems of supervision, mentoring, and monitoring of quality improvement - all areas reported as inadequate by all 11 country teams.

**Figure 5:**
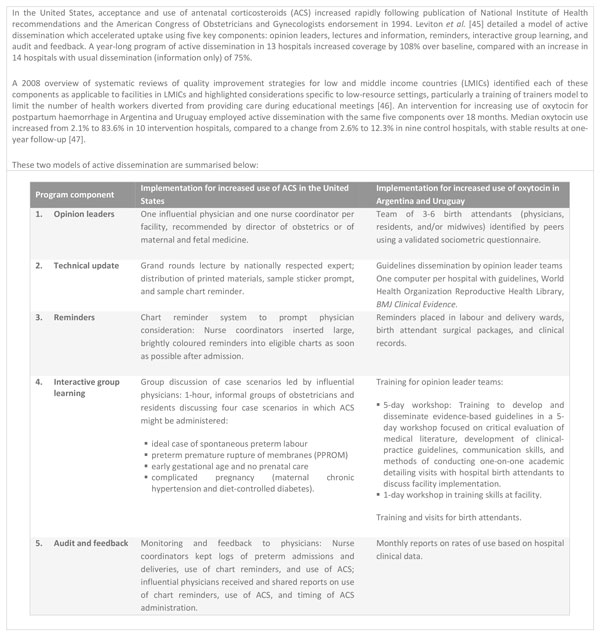
**An adaptable model for active dissemination of guidelines on the use of antenatal corticosteroids**. ACS: antenatal corticosteroids. LMICs: low and middle income countries

**Figure 6:**
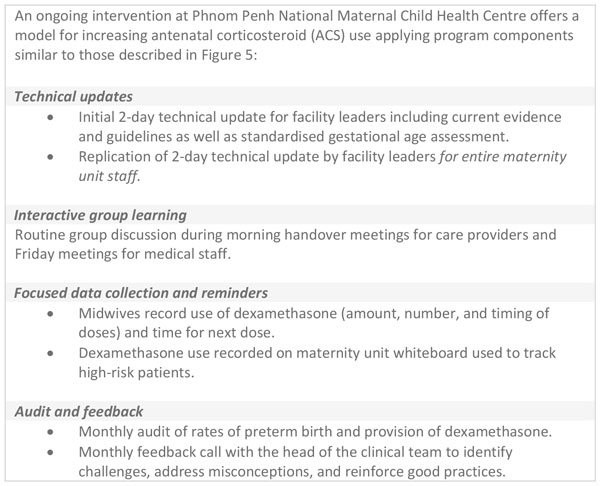
**Active dissemination of guidelines on the use of antenatal corticosteroids in Phnom Penh, Cambodia**. ACS: antenatal corticosteroids. LMIC: Low and middle income countries

A study of active dissemination in the United States showed that use of local opinion leaders, technical updates, reminders, interactive small-group learning, and audit and feedback were effective in accelerating facility uptake of ACS. These five elements were also effective in increasing use of a maternal health intervention in LMICs, with a structure reflecting the differing needs of low-resource settings. Figure 5 shows the five-component structure of the interventions in these two RCTs and details their implementation in each setting. Figure 6 outlines a program with similar features currently being studied in a facility in Cambodia. In particular, the audit and feedback component provides one model for continuous quality monitoring and improvement, an essential part of quality implementation of guidelines. This pilot model also provides a further example of the role of the continuous use of outcome data in improving safety and quality of care.

### Essential medical products and technologies priority actions

Corticosteroids for ACS treatment are inexpensive (often under 1 USD for a full course) and are often assumed to be widely available. Yet most respondents indicated shortages at the country level, primarily attributed to a lack of supporting policy or effective logistics systems. NEML listing for the fetal lung maturation indication is essential to prioritisation for procurement as well as integration into supply chain, from forecasting to distribution.

Adequate procurement further depends on development and dissemination of guidelines on ACS use, including clarity on the choice of corticosteroid and appropriate regimen. Two corticosteroids, dexamethasone and betamethasone, have been shown to be safe and effective to manage preterm birth. Betamethasone is sometimes preferred in HICs due to limited evidence suggesting better outcomes (44% reduction in RDS and 33% reduction in neonatal mortality versus placebo or no treatment, compared with 20% reduction in RDS and 28% reduction in neonatal mortality for dexamethasone versus placebo or no treatment) and potentially lower risk of maternal infection [4].

However, evidence on dexamethasone still conclusively supports its overall safe and effective use for management of preterm birth. A meta-analysis of 9 studies directly comparing dexamethasone with betamethasone further found no statistically significant differences apart from a greater reduction in intraventricular haemorrhage using dexamethasone (RR 0.44, 95% CI 0.21 to 0.92, 4 studies, 549 infants). A large RCT directly comparing dexamethasone to betamethasone is currently underway [32].

Critically, only dexamethasone is a feasible choice for scale-up in most LMICs. While dexamethasone is widely available from international suppliers for a variety of indications, the formulation of betamethasone supported by most evidence (a suspension of betamethasone acetate in betamethasone phosphate, rather than betamethasone phosphate alone) has been subject to global shortages [33] and costs 25 times as much as dexamethasone per course of ACS [34].

Dexamethasone also faces fewer policy hurdles to increased use for management of preterm birth. With a variety of uses, dexamethasone in the recommended formulation is already registered, listed on the NEML, and included in procurement and supply chain in all 11 countries, for other indications. Dexamethasone is the only ACS listed on the WHO EML with a fetal lung maturation indication [35].

National policies, in line with the current WHO guideline, should therefore focus on promoting appropriate use of dexamethasone through procurement policy, clear guidelines, and integration into forecasting, procurement, and supply chain.

### Limitations and implications for further research

Soliciting responses from a wide range of in-country partners and practitioners in maternal and newborn health captured context-specific challenges and generated collaborative solution ideas. The grading process also created consensus around priority bottlenecks and health system building blocks to be addressed. However, these consensus views are subjective. The quality and amount of information also varied depending on the level of knowledge of participants on health system issues and on workshop facilitation. In addition, bottlenecks were reported as perceived bottlenecks relative to the other health system building blocks. National-level assessment may mask regional disparities, particularly between urban and rural areas. This comprehensive questionnaire may have led to respondent fatigue, but this effect is least significant for ACS as the first intervention in the bottleneck analysis tool. For India and Pakistan, subnational responses may not mirror all national-level challenges. However, Pakistan submitted responses from all regions except two tribal territories, and although India returned data from only three of its 28 states, these areas are amongst the poorest states and include populations similar to those of Vietnam, Kenya, and DRC [36].

Further research is needed to establish the effectiveness of the solutions described here, especially in the context of ACS use in LMIC hospital settings where most of the world's births now occur. To date the evidence on ACS is primarily from HIC settings with neonatal intensive care [4] or the ACT trial from low-level settings with less-skilled workers [12]. The evidence base could be greatly advanced in LMIC settings, for instance through a multi-country, cluster-randomised trial in LMIC hospitals to assess the mortality impact and safety of a package optimising gestational age (where first trimester ultrasound is not routine) and clinical assessment of mothers, including risk of preterm birth and possible maternal infection, whilst providing appropriate maternal and newborn care. Given the lack of pharmacokinetic and dynamics data for both dexamethasone and betamethasone, other research questions include optimal dosage regimens. Data are now even more critically important and this gap is urgent to address, possibly for coverage and outcome data that could be tracked in routine health management information systems [29]. Further work is also needed to define signal functions for newborns by level of care. A signal function on ACS could be incorporated into existing monitoring systems, such as the signal functions for basic and comprehensive emergency obstetric care, to ensure consistent tracking of maternal and newborn interventions along the continuum of care [37].

**Figure 7:**
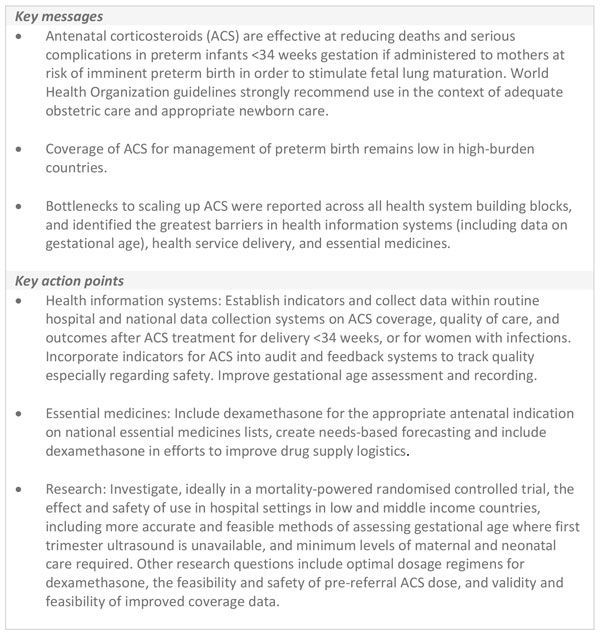
**Key messages and action points for scale-up of antenatal corticosteroids for management of preterm birth**. ACS: antenatal corticosteroids

Another important track of research is to improve the accuracy and also feasibility of gestational age assessment, including testing tools to improve accuracy of last menstrual period assessment, use of ultrasound dating in later pregnancy and ways to promote better recording in notes and use of the data by clinicians. Key messages and actions are summarised in Figure 7.

## Conclusions

ACS for management of preterm birth has been a standard of care in wealthy countries for over 20 years, yet is greatly underused in low-resource countries, where complications of preterm birth have recently become the leading cause of death in children under 5. Preterm infants are over 12 times more likely to die in the poorest compared to the richest countries, and infants in the poorest countries have a survival rate at 32 weeks similar to that of infants born in the richest countries at 25 weeks. This systematic bottleneck analysis using data from 11 countries identifies critical areas of focus and suggests a set of actionable solutions for extending this inexpensive, high-impact intervention, whilst also promoting the tracking for safety, potentially saving hundreds of thousands of lives each year.

## List of abbreviations

ACS: Antenatal corticosteroids; ANM: Auxiliary nurse-midwife; DRC: Democratic Republic of Congo; EML: Essential medicines list; FLM: Fetal lung maturation; HICs: High income countries; LIC: Low income country; LMIC: Low- or middle-income country; LMIS: Logistics management and information system; MIC: Middle-income country; MOH: Ministry of Health; NEML: National essential medicines list; NMR: Neonatal mortality rate; RCT: Randomised controlled trial; RDS: Respiratory distress syndrome; WHO: World Health Organization.

## Competing interests

The authors have not declared any competing interests. The assessment of bottlenecks expressed during consultations reflects the perception of the technical experts and may not be national policy. The authors alone are responsible for the views expressed in this article and they do not necessarily represent the decisions, policy or views of the organisations listed, including WHO.

## Authors' contributions

GL analysed data and drafted the manuscript in collaboration with JS. KED, AS-K JEL and SGM contributed to conceptualisation of the paper, data analysis and interpretation, and review of drafts. FA and MG and MM contributed to data interpretation and discussion and reviewed drafts. JMS, JH, PB, MJ, and EM contributed to country case studies. All authors reviewed drafts and approved the final manuscript.

## Supplementary Material

Additional file 1 Bottleneck tool questionnaire. Click here for file

Additional file 2 Supplementary tables, figures and literature search strategy. Click here for file

## Declarations

Publication costs for this supplement were funded by the Bill and Melinda Gates Foundation through a grant to US Fund for UNICEF (Grant ID: OPP1094117), and support from Save the Children's Saving Newborn Lives Programme. Additional funding for the bottleneck analysis was received from USAID (Grant ID: GHA-G-00-07-00007) through UNICEF.

This article has been published as part of *BMC Pregnancy and Childbirth *Volume 15 Supplement 2, 2015: Every Woman, Every Newborn. The full contents of the supplement are available online at http://www.biomedcentral.com/bmcpregnancychildbirth/supplements/15/S2.
