# Supplementary material for: Antenatal corticosteroids for management of preterm birth: a multi-country analysis of health system bottlenecks and potential solutions
Source: BMC Pregnancy Childbirth. 2015 Sep 11;15(Suppl 2):S3. doi: 10.1186/1471-2393-15-S2-S3 (PMC4577756; doi:10.1186/1471-2393-15-S2-S3)
Supplement: Additional file 2 — Supplementary tables, figures and literature search strategy. [file 1471-2393-15-S2-S3-S2.docx]

Antenatal corticosteroids for management of preterm birth: a multi-country analysis of health system bottlenecks and potential solutions

Additional file 2

A. Table S1 Reported bottlenecks to scale-up of coverage with antenatal corticosteroids, with direct causes where specified 2

B. Table S2: Bottlenecks (all countries) for antenatal corticosteroids by health system building block 4

C. Figure S1: Subnational grading of bottlenecks for antenatal corticosteroids for management of preterm birth 11

D. Table S3: National reported policies on allowed level(s) of care, authorised health provider cadre(s), and recommended drug for antenatal corticosteroids for management of preterm birth 12

D. Table S4: Subnational reported policies on allowed level(s) of care, authorised health provider cadre(s), and recommended drug for antenatal corticosteroids for management of preterm birth 13

E. Literature search strategy 14

# A. Table S1 Reported bottlenecks to scale-up of coverage with antenatal corticosteroids, with direct causes where specified

| Health system building block | Bottleneck | | Africa | | | | | | Asia | | | | |
| --- | --- | --- | --- | --- | --- | --- | --- | --- | --- | --- | --- | --- | --- |
|  |  |  | Cameroon | DRC | Kenya | Malawi | Nigeria | Uganda | Bangladesh | India^a^ | Nepal | Pakistan | Vietnam |
| Leadership and governance | Lack of awareness and strategy for preterm birth | |  |  |  | ✓ |  |  |  | b |  | ✓^c^ |  |
|  | Inadequate guidelines on ACS use | | ✓ | ✓ | ✓ |  | ✓ | ✓ | ✓ | ✓ | ✓ | ✓ |  |
|  |  | None |  | ✓ |  |  |  |  | ✓ | ✓ | ✓ | ✓^c^ |  |
|  |  | Outdated | ✓ |  |  |  |  |  |  |  |  |  |  |
|  |  | Lacking dissemination |  |  | ✓ |  | ✓ | ✓ |  |  |  | c |  |
| Health financing | Insufficient funding for ACS | |  | ✓ | ✓ | ✓ | ✓ | ✓ | ✓ | ⅓^b^ | ✓ | ✓^c^ |  |
|  |  | Due to lack of priority or policy |  |  | ✓ | ✓ | ✓ | ✓ | ✓ | ⅓^b^ | ✓ | ✓^c^ |  |
|  |  | Unspecified cause |  | ✓ |  |  |  |  |  |  |  |  |  |
|  | Patient bears cost of ACS | | ✓ | ✓ |  |  | ✓ |  | ✓ | ⅔ | ✓ | ✓ |  |
| Health workforce | Shortage of health workers managing women in preterm labour | | ✓ | ✓ | ✓ | ✓ | ✓ | ✓ | ✓ | b | ✓ | ✓^d^ | ✓ |
|  | Lack of training on recognition of preterm labour and management with ACS | | ✓ | ✓ | ✓ | ✓ | ✓ | ✓ | ✓ | ✓ | ✓ | ✓^c^ | ✓ |
|  | No job descriptions or job aids for management of preterm labour using ACS | |  | ✓ | ✓ |  | ✓ | ✓ | ✓ | ✓ | ✓ | ✓^c^ |  |
|  | No or inadequate supervision and mentoring | | ✓ | ✓ | ✓ | ✓ | ✓ | ✓ | ✓ | ✓ | ✓ | ✓ | ✓ |
| Essential medical products | NEML includes dexamethasone but lacks fetal lung maturation indication (for any corticosteroid) | | ✓ | ✓ | ✓ | ✓ | ✓ | ✓ | ✓ | ✓ | ✓ | ✓ | ✓ |
|  | Inadequate procurement or distribution of ACS | |  |  | ✓ | ✓^e^ | ✓ | ✓ | ✓ |  | ✓ | ✓ | ✓ |
|  |  | Due to lack of policy or NEML indication |  |  | ✓ | ✓ | ✓ | ✓ |  |  | ✓ | ✓ |  |
|  |  | Due to slow procurement process |  |  |  |  |  |  | ✓ |  |  |  |  |
|  |  | Due to lack of market availability |  |  |  |  |  |  |  |  |  |  | ✓ |
|  | No forecasting and no or inadequate logistics management information systems | | ✓ | ✓ | ✓ | ✓ | ✓ | ✓ | ✓ | ✓ | ✓ | ✓ | ✓ |
| Health service delivery | Health workers who manage women in preterm labour not permitted to prescribe ACS | |  | f | ✓ | ✓ | ✓ | ✓ | ✓ | ✓ | ✓ | ✓ | ✓ |
|  | No or inadequate quality monitoring and improvement programs | | ✓ | ✓ | ✓ | ✓ | ✓ | ✓ | ✓ | ✓ | ✓ | ✓ | ✓ |
|  | Delays due to referrals | | NR | NR | ✓ | ✓ | ✓ | ✓ | NR | ✓^f^ | f | ✓ | ✓ |
| Health information system | No indicators for ACS use | | ✓ | ✓ | ✓ | ✓ | ✓ | ✓ | ✓ | ✓ | ✓ | ✓ | ✓ |
|  | No critical review of ACS use in clinical audits or perinatal death reviews | | ✓ | ✓ | ✓ | ✓ | ✓ | ✓ | ✓ | ✓ | ✓ | ✓ | ✓^g^ |
|  | ACS use not included in birth records or safe childbirth checklists | | ✓ | ✓ | ✓ | ✓ | ✓ | ✓ | ✓ | ✓ | ✓ | ✓ | ✓ |
|  | ACS use not included in partographs or Safe Birth checklist | | ✓ | ✓ | ✓ | ✓ | ✓ | ✓ | ✓ | ✓ |  | ✓^c^ | ✓ |
| Community ownership and partnership | Lack of awareness and programs targeting awareness of ACS and preterm birth | | ✓ | NR | ✓ | ✓ | ✓ | ✓ | ✓ | ✓ | ✓ | ✓ | h |
|  | Transportation or geographical access | | ✓ | NR | ✓ |  | ✓ | ✓ | NR | ✓ | ✓ | ✓ | ✓ |
|  | Gender issues, female disempowerment, and lack of male involvement | |  | ✓ | ✓ |  | ✓ | ✓^i^ | NR | ✓ | ✓ | ✓ | ✓^j^ |

^a^ Not all states responded completely to survey questions; for mixed responses, the number of states reporting a bottleneck is shown as a fraction

^b^ Question missing from survey

^c^ Except Sindh province

^d^ Azad Jammu and Kashmir and Sindh province; no response from Baluchistan province or Gilgit-Baltistan

^e^ 17% uninterrupted availability over 12 months preceding survey

^f^ No policy

^g^ No clinical audits or perinatal death reviews in Vietnam

^h^ Generally high awareness except among ethnic minority groups

^i^ Domestic violence and lack of female financial and decision-making autonomy

^j^ Especially among ethnic minority groups

NR= no response

ACS= antenatal corticosteroids

NEML= national essential medicines list

LMIS= logistics management information system

# B. Table S2: Bottlenecks (all countries) for antenatal corticosteroids by health system building block

| **Health system Building Block** | **Bottlenecks** |
| --- | --- |
| **Leadership and governance** | - Road map and child survival and development strategic documents mention use of corticosteroids but health workforce have not been trained and sensitised on the same. - Fetal lung maturation is an indication for dexamethasone in the 2010 Clinical guidelines, but this is not widely disseminated. - Interventions for preterm not implemented. - Standards and guidelines poorly disseminated. - Only physicians/doctors can prescribe antenatal steroids as a matter of policy. - Lack of updated normative documents with the integration of antenatal corticosteroids in the normative documents. - No national standard treatment guidelines or clinical protocols on the use of antenatal corticosteroids (ACS) for fetal lung maturation in place.      - No existing guideline and protocols for antenatal corticosteroid administration for prevention of preterm birth. - No government policy for the use of antenatal corticosteroids exists for lung maturation of preterm and kangaroo mother care for low birth weights. - No National standard treatment guidelines or clinical protocols covering prevention and management of preterm birth. - True incidence of prematurity is a cause of neonatal deaths not realised, figures of corticosteroids in practice. Lack of awareness and realisation. - Although there are targets on newborn mortality reduction but still no specific professional guidelines to achieve the specified targets, including the use of prenatal corticoid. - Need to strengthen leadership on pregnancy management for early detection of newborn at risk of prematurity for early prevention by corticosteroid. - Management of preterm birth is not well covered in reproductive maternal, newborn and child health strategy and guidelines. - Intervention is mentioned in the Malawi Standard Treatment Guidelines (MSTG) but not covered in the clinical protocols. |
| **Health financing** | - The indirect costs to come to hospital are not insignificant. Mothers come too late to be able to receive ACS. - Inadequate financial resources. - Dexamethasone is cheaper than betamethasone. - The cost and availability of antenatal corticosteroid. - The purchase of the drug is not budgeted for by the facilities. - Patients still has to pay for the drug on getting to the hospital. - Majority of Nigerians are not covered by the Insurance Health System. - For the few with the Insurance, the insurance policy does not cover the antenatal steroids drug, as is not part of the routine antenatal drugs. - Pregnant women pay for care (prenatal and postpartum) except vaccination against tetanus, intermittent preventive treatment and antiretroviral therapy. - Existence of financial barriers to assess maternal and newborn care. - Insufficient funding of the state for newborn care. - Dependence of partner support. - Financial inaccessibility for families. - Budget for Antenatal corticosteroid is not yet included in any of the operational plans of Health, Population and Nutrition Sector Development Program (2011-2016). - Lack of financing-based programmes. - Financial implications have not been weighed in under this intervention because corticosteroids have not been incorporated national protocols and program. - Funding system is not specific for specified drugs and supplies. - Funds are inadequate to have consistent supply of corticosteroids as it is with other supplies. |
| **Health workforce** | - Limited health workers knowledge on the use of the drug. - Lack of staff training on the management of cases of prematurity. - Lack of human resources capacities to recognise manage preterm births. - Inadequate health workforce (shortage of obstetrics and gynaecology specialist at health facility). - Shortage of corticosteroid used for pregnant women with risk of premature birth due to these medicines not available in the market. - No adequate numbers of health workforce who can use the drug/only clinicians and specifically doctors who prescribe. - Training on the standardised use of the antenatal steroids does not exist for the physicians and all health workers including the private physicians. - No competency-based training. - Inadequate numbers of competently trained health workers in all cadres of health facilities. - Inadequate and inconsistent training for all cadres of health workers. - Knowledge issue for most health workers. - Job aids that do not address antenatal corticosteroids. - Health staff is empowered to administer corticosteroids but are not forms / recycled, there is no mechanism for monitoring/control. - Lack of qualified staff motivated and competent; lack of midwives. - Limited access and availability of skilled providers. - Absence of guidelines and protocols. - No specific job descriptions, training/orientation related to use of antenatal corticosteroid. - Sufficient work force not trained and skilled on the use of natal use of corticosteroids due to lack of policy framework. - No (standard) job descriptions for health workers for all levels of care, which reflect their role in prescribing and /or administering antenatal corticosteroids. - Authorisation of protocol medications prescription and administration. - Job descriptions for health workers reflecting their role prescribing and/ or administering antenatal. - Inadequate funding for training and monitoring and evaluation. - Inadequate capacity, not received updated information and skills. - Systems for Mentorship and supervision programs are not efficient. - The drug is not on the list to be prescribed and administered by nurse/midwives when need arise. |
| **Essential Medical Products and Technologies** | - Supply chain problems- forecasting and quantification (Skills). - The drug antenatal steroids are not in the Essential drug List. - The central procurement process is time consuming. - Antenatal corticosteroids not included in essential drug list. - It is in national essential medicines list, but not indicated for fetal lung maturation. - Dexamethasone is there but not in use at lower levels for fetal lung maturity. - Gaps in forecasting and quantification. - No country wide use of corticosteroids for fetal lung maturity. - It is in the national essential medicines list, but it is an "Essential" drug, not "Vital" within the vital essential and non-essential list of drugs system. - There are problems with the push and pull systems, but we do not think it is a supply chain issue but rather and end- user issue. - Drugs are used inconsistently by institutions mainly tertiary. - No state or local systems in place to forecast accurately the need for antenatal corticosteroids medicine. - Although, corticosteroids are available at the health care system (and in market) but rarely used at the antenatal period for enhancing fetal lung maturation. - No indication system for shortfalls in availability; inadequate distribution system. - The current drug list of Vietnam does not list antenatal corticoid for lung maturation in premature management. However, there may be corticosteroids (branded or generic) listed, which could be used for lung maturation. - Analysis has showed 17% uninterrupted corticosteroids availability but not specific for preterm birth. - Possibly the drugs are not on the list of essential drugs, and there is evidence on inconsistent availability of the products. |
| **Health service delivery** | - Pilot studies need to be widely disseminated and scaled-up. - No protocols/standard operating procedures/job aids in place and clear implementation policies. - Use depends on knowledge and willingness of senior staff. - There are no protocols for who can give ACS so it is not widely used during preterm labour. - Limited awareness and sensitisation at the level of service delivery. - No quality improvement programme for antenatal corticosteroid. - There is fragmentation at all levels with greater fragmentation of care at the lower level. - Barrier to antenatal corticosteroids: Lack of awareness and ignorance. There is a risk of misuse and overuse. - Staff unaware and not trained on the antenatal use of corticosteroids. - Attitudes of health workers may be a problem. - Timely identification of the problem. - Timely administration of ACS. - Antenatal corticosteroids has not been incorporated in health care delivery system. - Reproductive health standards available but not consistently used. - Unavailability of the service at district level facility and primary level where preterm births occur. - Knowledge gap to identify women in need of the service. - Ineffective referral system for women to access preterm management at a specific level of care. - Restrictions on prescription of drugs to other service providers. - Ineffective birth preparedness. |
| **Health Information system** | - No indicators captured because ACS is not routinely used (Prescribed only by clinicians and mainly by obstetricians/Gynaecologists). - We do not have any information on who is giving ACS and where. - No place or guidance for what to record, e.g. on passport or partograph. - No indicators used to assess coverage of antenatal corticosteroid. - No awareness of reporting on the use of antenatal corticosteroids. - Kangaroo mother care and ACS are not included perinatal death audits. - Use of steroid is not included in any birth record or district health information systems. - Too many logbooks, forms, records, overload to health workers, leading to inaccurate data. - No data available on preterm management. - Data records are not comprehensive enough to trace use of corticosteroids. - Record keeping for case files is not effective that one cannot easily trace use of corticosteroids. |
| **Community ownership and partnership** | - Not friendly information, education and communication material – language. - Social cultural factors. - Long waiting hours at the facility. - Access to the facility. - No community units. - Dormant community units. - The effort for promoting community demand is not equal across the country. - Barriers exist to seeking care including indirect/ opportunity costs and some socio- cultural issues. - Women seek care too late even if they are at high risk (e.g. previous preterm). - Patriarchal society therefore means that health services are delayed. - Lack of affordable transportation to service delivery point.      - Poverty. - Evidence based interventions are most times in conflict with traditional practices and therefore not easily accepted hence the need for a lot behaviour change communication. - Low involvement of men in issues of maternal and child health. - Community groups and Community support groups for the operating community clinics exist but their awareness and activities are limited especially related to maternal newborn health services. - Transportation options to medical care facilities are inadequate. - Socio-cultural barriers to use of services exist. - Community ownership and partnership is very limited. - Community lacks information on maternal child health. - People consider maternal child health is just a responsibility of health workers. - Limited communication skills of the health communicators. - Antenatal care coverage is good but quality and comprehensiveness of the care provided is very low. - Information giving to communities is generalised not specific to a topic of interest. |

# C. Figure S1: Subnational grading of bottlenecks for antenatal corticosteroids for management of preterm birth

# D. Table S3: National reported policies on allowed level(s) of care, authorised health provider cadre(s), and recommended drug for antenatal corticosteroids for management of preterm birth

|  | |  |  |  |  |  |  |  |  |  |
| --- | --- | --- | --- | --- | --- | --- | --- | --- | --- | --- |
|  | | **Country** | | | | | | | | |
|  | | Cameroon | DRC | [Kenya](#RANGE!D31)^a^ | Malawi | Nigeria | Uganda | Bangladesh | Nepal^a^ | Vietnam |
| Allowed level(s) of care | Tertiary | ✓ | No Policy | ✓ | ✓ | ✓ | ✓ | No policy | ✓ | ✓ |
|  | Secondary | ✓ |  | ✓ | ✓ |  | ✓ |  |  | ✓ |
|  | Primary |  |  |  |  |  | PRE |  |  |  |
| Health worker cadres authorised to prescribe and administer | Nurse | P-A | No Policy | A | A | A | A | A | A | A |
|  | Midwife | P-A |  | A | A | A | A/P-A^e^ | A | A | A |
|  | Associate clinician | P-A |  | P-A |  |  | P-A |  | A | A |
|  | Non-specialist doctor | P-A |  | P-A | P-A | P-A | P-A | P-A | A | P-A |
|  | Specialist doctor/other | P-A^b^ |  | P-A^c^ | P-A^d^ | P-A | P-A | P-A | P-A^f^ | P-A |
| Preferred or main drug(s) | Dexamethasone |  | Unspecified | ✓ | No response | Unspecified | No response | ✓ | ✓ | ✓ |
|  | Betamethasone | ✓ |  |  |  |  |  |  |  | ✓ |

a No official policy on allowed level(s) of care

b Auxiliary obstetricians

c Medical officers and clinical officers

d Clinical officers

e Midwives can prescribe and administer in Ugandan private sector

f Registered doctors

PRE= pre-referral dose only; P-A= prescribe and administer; A= administer only; Unspecified= responded to questions about preferred drug(s) without specifying

# D. Table S4: Subnational reported policies on allowed level(s) of care, authorised health provider cadre(s), and recommended drug for antenatal corticosteroids for management of preterm birth

|  | | |  |  |  |  |  |  |  |  |  |
| --- | --- | --- | --- | --- | --- | --- | --- | --- | --- | --- | --- |
|  | | | **India^a^** | | | **Pakistan^b^** | | | | | |
|  | | | Andhra Pradesh | Odisha | Rajasthan | Balochistan | KPK | Punjab | Sindh | AJK | Gilgit-Baltistan |
| Allowed level(s) of care | Tertiary | | No policy | ✓ | No policy | ✓ | ✓ | ✓ | ✓ | ✓ | ✓ |
|  | Secondary | |  |  |  |  |  |  | ✓ |  | ✓ |
|  | Primary | |  |  |  |  |  |  | PRE |  |  |
| Health worker cadres authorised to prescribe and administer | Nurse | | No policy | A | No policy | A | A | A | A | A | P-A |
|  | Midwife | |  | N |  | A |  |  | P-A | A | A |
|  | Associate clinician | |  | A |  | P-A | P-A | P-A | P-A | A | P-A |
|  | Non-specialist doctor | |  | P-A |  | P-A | P-A | P-A | P-A | P-A | P-A |
|  | Specialist doctor/other | |  | P-A | P-A^c^ | P-A | P-A | P-A | P-A | P-A | P-A |
| Preferred or main drug(s) | Dexamethasone | | Not asked | Not asked | Not asked | No response | No response | No response | Unspecified | Unspecified | Unspecified |
|  | Betamethasone | |  |  |  |  |  |  |  |  |  |

India has since adopted national policy allowing pre-referral prescription and administration by auxiliary nurse-midwives at any level of care

b KPK = Khyber Pakhtunkhwa, AJK = Azad Jammu and Kashmir

c Medical officers authorised but not practicing

PRE= pre-referral dose only; P-A= prescribe and administer; A= administer only; N= no authorisation; Unspecified= responded to questions about preferred drug(s) without specifying

# E. Literature search strategy

**For the background and discussion sections, we used the following search terms in Pub Med and the Cochrane Library. Limits were applied and only relevant articles were retrieved.**

**Newborn**

(neonat* OR newborn* OR new-born* OR infant)

AND

**Antenatal corticosteroids**

(Antenatal OR prenatal) AND (corticosteroids OR steroids OR dexamethasone OR betamethasone)

**For the discussion section we searched the following terms in Pub Med. Only relevant articles were retrieved.**

**Newborn**

(neonat* OR newborn* OR new-born* OR infant)

AND / OR

**Coverage, bottlenecks, and solutions**

(coverage OR use)

AND / OR

**Bottlenecks**

(bottlenecks OR barriers OR challenges)

AND / OR

**Solutions**

(scale-up OR scale up OR scaling up OR increasing use OR solutions)
